# Modulating the resting-state functional connectivity patterns of language processing areas in the human brain with anodal transcranial direct current stimulation applied over the Broca’s area

**DOI:** 10.1117/1.NPh.5.2.025002

**Published:** 2018-02-27

**Authors:** Jianwei Cao, Hanli Liu, George Alexandrakis

**Affiliations:** University of Texas at Arlington and University of Texas Southwestern Medical Center at Dallas, Joint Graduate Program in Biomedical Engineering, Arlington, Texas

**Keywords:** functional near-infrared spectroscopy, transcranial direct current stimulation, language cortex, resting-state functional connectivity

## Abstract

Cortical circuit reorganization induced by anodal transcranial direct current stimulation (tDCS) over the Broca’s area of the dominant language hemisphere in 13 healthy adults was quantified by functional near-infrared spectroscopy (fNIRS). Transient cortical reorganization patterns in steady-state functional connectivity (seed-based and graph theory analysis) and temporal functional connectivity (sliding window correlation analysis) were recorded before, during, and after applying high current tDCS (1 mA, 8 min). fNIRS connectivity mapping showed that tDCS induced significantly (p<0.05) increased functional connectivity between Broca’s area and its neighboring cortical regions while it simultaneously decreased the connectivity to remote cortical regions. Furthermore, the anodal stimulation caused significant increases to the functional connectivity variability (FCV) of remote cortical regions related to language processing. In addition to the high current tDCS, low current tDCS (0.5 mA, 2 min 40 s) was also applied to test whether the transient effects of lower stimulation current could qualitatively predict cortical connectivity alterations induced by the higher currents. Interestingly, low current tDCS could qualitatively predict the increase in clustering coefficient and FCV but not the enhancement of local connectivity. Our findings indicate the possibility of combining future studies fNIRS with tDCS at lower currents to help guide therapeutic interventions.

## 1 Introduction

Transcranial direct current stimulation (tDCS) is a noninvasive brain stimulation technique that has been applied to modulate cognitive function so as to enhance performance in healthy subjects^1^^–^^3^ and facilitate neurorehabilitation during stroke recovery.^4^ Typically, tDCS is used with the intention to alter cortical excitability by delivering weak currents (1 to 2 mA) through a pair of anode–cathode electrodes for 8 to 20 min.^5^ The primary mechanisms of the excitability shifts are depolarization of resting membrane potential by anodal stimulation and hyperpolarization of resting membrane potential by cathodal stimulation, as has been illustrated in animal studies.^6^^,^^7^ In the context of human subject studies on areas of the brain controlling language processing, which is the focus of this work, it has been suggested that anodal tDCS over either Broca’s area or Wernicke’s area can improve naming accuracy or speed both in stroke-induced aphasia patients^8^^–^^10^ and in healthy subjects.^3^^,^^11^^,^^12^

Despite the above-described growing number of studies investigating how tDCS affects language performance through stimulation of language processing areas, there has been relatively little study of how tDCS affects cortical functional connectivity reorganization during those interventions. Meinzer et al.^13^ used resting-state functional magnetic resonance imaging (fMRI) to assess the impact of anodal tDCS on the functional connectivity networks of healthy subjects. Their results showed an increase of functional connectivity strength in language-associated regions, such as dorsolateral and medial prefrontal regions, presupplementary motor area (pre-SMA) and SMA but a decrease in more posterior and occipital regions. Marangolo et al.^14^ revealed that increased connectivity strength was most pronounced in the left hemispheric structures related to planning, maintenance, and execution of speech. They used resting-state fMRI to study the effects of bilateral tDCS on aphasia patients, with the anodal patch applied over the Broca’s area of the lesion-containing left brain hemisphere and the cathodal patch applied over the homologous contralateral brain region. Also, the presently few studies of tDCS-mediated changes on connectivity have focused on steady-state rather than the temporal aspects of those changes. The fact that tDCS could affect dynamic fluctuations in functional connectivity has not yet been explored even though the study of these fluctuations has been shown to elucidate fundamental properties of brain networks.^15^^–^^18^

One additional aspect of tDCS studies that remains underexplored to date is the effect of electrode placement on language performance. For example, Monti et al.^19^ found out that anodal tDCS over the left Broca’s area did not improve naming accuracy in chronic nonfluent aphasic patients, whereas Baker et al.^8^ reported improved naming performance for stimulation over the same area. It is conceivable that due to heterogeneity of lesion effects in aphasic patients, an optimal tDCS electrode placement for language enhancement and recovery would differ between subjects. In an ideal scenario, investigators would be able to try different tDCS electrode placements rapidly to find the one that could maximize performance improvements in each patient. However, the current intensities used in tDCS interventions produce long-lasting effects on cortical activity with durations from hours to days,^20^ which makes the rapid testing of multiple electrode placements impossible. One potential way to reduce testing time could be to use lower tDCS currents, which reduce the duration of tDCS effects on the brain hemodynamics to a few minutes. We have previously shown that use of lower tDCS currents allows multiple tDCS placements to be tested within one single session.^21^ However, the potential utility of lower current tDCS depends on whether it could produce qualitatively similar patterns of connectivity change to those seen during the high current tDCS condition.

This work is a first step toward addressing the aformentioned knowledge gaps in tDCS studies, namely (1) to study the effect of stimulation current on the steady-state and temporal connectivity changes in brain networks related to language processing and (2) to study whether lower current tDCS patterns could produce changes in connectivity patterns that are qualitatively similar to those observed during and after the application of larger tDCS currents. Here, we have studied the effect of tDCS current intensity on the cortical connectivity maps of healthy adults by use of functional near-infrared spectroscopy (fNIRS). fNIRS can detect changes in the concentration of oxyhemoglobin (HbO) and deoxyhemoglobin (Hb) resulting from neurovascular coupling^22^ and has been increasingly shown to be a viable alternative neuroimaging modality to fMRI despite its lower spatial resolution and ability to only map cortical activation.^23^^,^^24^ Also, the compatibility of fNIRS with tDCS makes it advatangeous for studying brain reorganization induced during tDCS.^25^ Furthermore, fNIRS provides higher temporal resolution relative to fMRI, which is valuable for investigating time-varying functional connectivity in the brain.^26^

Several fNIRS-tDCS studies have been reported in the past years. Merzagora et al.^27^ stated that a significant HbO increase was observed by fNIRS over a prefrontal cortex (Fp1) stimulation site for 1 mA of anodal tDCS applied for 8 min. Takai et al.^28^ found that both anodal and cathodal stimulation of 1 mA for 20 min applied to the primary motor cortex (M1) induced a significant HbO decrease in the contralateral premotor cortex, SMA, and M1. Our group^25^ revealed that bihemispheric tDCS with anode on the left M1 and the cathode on the right M1 changed resting-state connectivity from intrahemispheric to interhemispheric and increased flexion speed when subjects performed a wrist flexion task. However, to our knowledge, no fNIRS studies to date have reported how tDCS affects the reorganization of language area networks. Specifically, in this work, we wanted to assess the alterations of functional connectivity due to anodal tDCS over the left Broca’s area and whether a low tDCS current (0.5 mA) could qualitatively predict cortical connectivity patterns occurring after a high tDCS current (1 mA), which is a standard intervention current choice in the literature. Cortical connectivity patterns were computed using seed-based functional connectivity with the seed located on the anodally stimulated left Broca’s area and on its homologous area on the right hemisphere. Furthermore, we computed changes in time-variant functional connectivity with the same seeds and also employed graph theory analysis to assess global pattern changes with tDCS current intensity. Cortical reorganization patterns of functional networks created in response to low current tDCS were compared to those during and after high current tDCS. The findings of this work are discussed in light of their potential future utility for helping guide therapeutic interventions.

## 2 Methods and Materials

### 2.1 Subjects

Thirteen right-hand subjects (2 females, 11 males, mean±SD age=35.4±8.4) were recruited in this study. Subject handedness was determined by the Edinburgh Handedness Inventory.^27^ All subjects were healthy and did not have a history of neurological disorders. Written informed consent was obtained from each participant before the experiments. The studies were carried out under the approval of the University of Texas at Arlington Institutional Review Board protocol (UTA #2015-0819).

### 2.2 fNIRS Imaging Setup Combined with tDCS

**Fig. 1:**
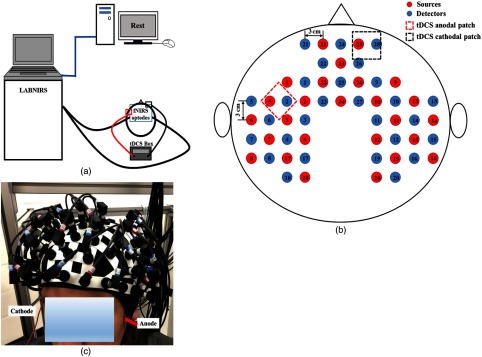
(a) A schematic representation of the overall instrumentation setup. The protocol only with “rest” is shown on the screen. FNIRS optodes and tDCS electrodes were placed on the subject’s head, as described in Sec. 2.2. (b) Schematic of the fNIRS probe geometry with 26 sources and 28 detectors placed over a subject’s head. The separation of all source and detectors is 3 cm (red dots: sources, blue dots: detectors). (c) Placement of the fNIRS-tDCS assembly on a subject’s head. The gray arrow points to the wire connecting the cathode patch and the red arrow points to the wire connecting the anode patch.

Figure 1(a) illustrates the overall instrumentation setup. A continuous-wave fNIRS imaging system (LABNIRS, Shimadzu, Japan) was used, which utilized near-infrared light diode sources (at wavelengths of 780, 805, and 830 nm) and photomultiplier detectors. A schematic of the fNIRS source–detector geometry is shown in Fig. 1(b). The setup geometry consisted of 26 sources and 28 detectors with a separation of 3 cm, resulting in 83 source–detector channels, which were inserted into the optode holder on the subject’s head. This probe geometry covered cortical areas known to be part of the language network, including the Broca and Wernicke’s areas of the left hemisphere and their homologous locations in the right hemisphere, as well as some prefrontal cortical regions, including the frontopolar, dorsolateral prefrontal cortex, and premotor areas. fNIRS signals were sampled at a frequency of 12.35 Hz.

**Fig. 2:**
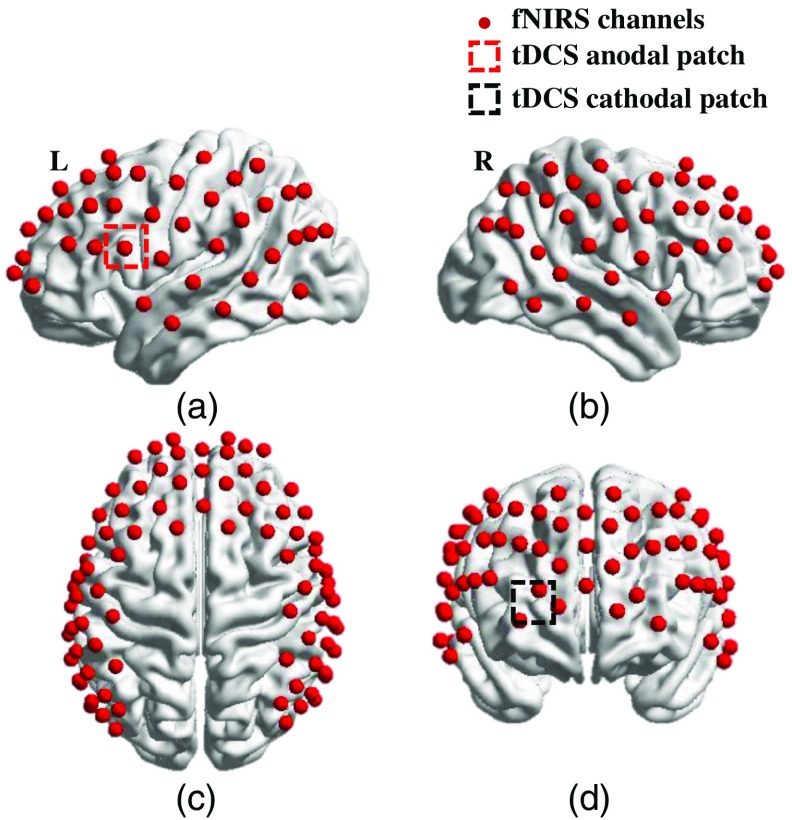
Coregistration of fNIRS source-detector channels (mid-way points between source and detector pairs) on a standard human brain atlas: (a) Sagittal view (left), (b) sagittal view (right), (c) top view, and (d) coronal view. tDCS anodal patch (red dashed square) and cathodal patch (black dashed square) are placed on the left FC5 position (a) and right Fp2 position (d).

A coregistration procedure was performed based on cranial landmark measurements on all the subjects^23^ to estimate the cortical regions covered by the fNIRS source–detector geometry. A motion tracking system (Fastrak, POLHEMUS) was used to measure five reference cranial landmarks (nasion, inion, left and right preauricular points and vertex), as well as the location of all source and detector optodes. The Montreal Neurological Institute coordinates were calculated from the real-world stereotaxic coordinates of the optodes with the five cranial landmarks as reference positions.^29^ NIRS Statistical Parametric Mapping^30^ was used to register the optodes on the standard MRI brain template and identify the Brodmann areas of each source–detector channel. Figure 2 shows the spatially registered channels (averaged over all 13 subjects) on the standard human brain atlas.

tDCS current was applied by a battery-driven electrical stimulator (Phoresor II, IOMED Inc., Salt Lake City, Utah) connected by a pair of saline-soaked gauze covered gel electrodes (5×5  cm; IOMED Inc., Salt Lake City). The tDCS patches were placed on the head using the EEG International 10/20 system^31^ as a reference. The anodal patch was placed over the FC5 position to stimulate left Broca’s area, which is centered at channel 27 in our setup, and a cathodal patch was centered over the Fp2 position^12^ as control [dashed square in Figs. 1(b), 2(a), and 2(d)]. Two 0.5-cm-diameter holes were punctured on each patch to fit through optical fiber bundles that overlapped spatially with the patches.

### 2.3 Protocol Design

**Fig. 3:**
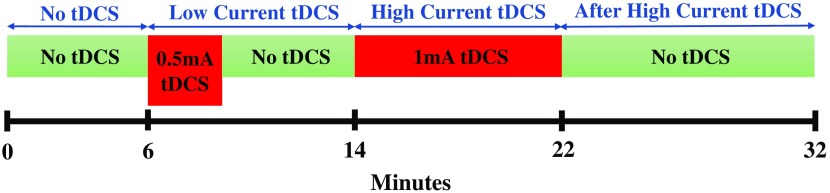
The tDCS protocol timeline.

Subjects were asked to rest for 32 min with eyes closed but awake while fNIRS measurements were performed. The first 6 min before tDCS were used as baseline, and then 0.5-mA current tDCS, henceforth referred to as low current tDCS, was applied for 2 min 40 s. Subsequently, subjects were asked to continue to rest for 5 min and 20 s, which was ample time for any hemodynamic signatures of the low current tDCS to disappear^5^^,^^21^^,^^32^ while also maintaining a practically feasible total protocol duration. After that, a higher current of 1 mA, henceforth referred to as high current tDCS, was applied for 8 min. These current intensity and stimulation duration settings were selected to be near the lower bound of values used in current clinical interventions involving tDCS in order to minimize protocol duration and improve subject comfort. The fNIRS imaging session concluded with 10 min of acquiring data while subjects rested so as to record changes in the hemodynamics immediately after the end of the 1-mA tDCS. A schematic of the timeline of the protocol is shown in Fig. 3. All subsequent fNIRS data analyses were performed separately for each one of the four stimulation conditions: no tDCS (0 to 6 min), low current tDCS (6 to 14 min), high current tDCS (14 to 22 min), and after high current tDCS (22 to 32 min). The low current tDCS session composed of the sum of 2 min 40 s of 0.5 mA followed by 5 min and 20 s of no tDCS. It was found necessary to merge those two periods into one, combining signals measured during low current tDCS and its subsequent decay to baseline hemodynamics, in order to attain sufficient fNIRS time-series data to enable all subsequent functional connectivity analyses for this stimulation condition. Subjects were not told when tDCS was applied.

### 2.4 Data Preprocessing

This study used a publically available toolbox named Homer^33^ to process the time-series fNIRS data. First, the data from each channel were band-pass filtered (0.009 to 0.1 Hz) to reduce low-frequency drift and high-frequency neurophysiological noise.^34^^,^^35^ Subsequently, changes in HbO relative to the baseline were quantified by a modified Beer–Lambert’s law.^35^ Correlation-based signal improvement^36^ was adopted to remove motion artifacts based on negative correlation between oxygenated and deoxygenated hemoglobin dynamics.

### 2.5 Data Analysis

#### 2.5.1 Seed-based functional connectivity

Channels 27 and 34 were chosen as seeds. Channel 27 was located over the stimulated left Broca’s area, which is the dominant language area for right-handed subjects. Channel 34 was located over the right Broca’s homologue, which is known to endow verbal communication with additional meaning by contributing to the analysis of emotional and tonal context in language.^37^ Pearson’s correlation coefficients were calculated between the seed channel and all the other channels for the entire time interval allocated to each stimulation condition (Fig. 3). Subsequently, a Fisher’s z-transformation was employed to transform the correlation coefficients to z-values^38^ for each subject. For each stimulation, session z-values between all the other channels and the seed channel were averaged across subjects. Significant seed-based functional connectivity changes between stimulation sessions were identified based on the following criteria:^39^ (1) using a paired t-test, for each z-value between sessions (i.e., no tDCS versus low current tDCS) at a threshold of uncorrected p<0.05 and (2) z-values were significantly different from zero at p<0.05 significance using a one-sample t-test in at least one session. The significant changes in functional connectivity were mapped by BrainNet Viewer software.^40^

#### 2.5.2 Time-variant functional connectivity

Time-variant functional connectivity was also calculated for the same seed locations. For each individual subject, sliding-window correlation (SWC) analysis^15^^,^^41^ was performed on each one of the four session data. In our SWC analysis, a 60-s time window was selected and then shifted by an increment of 1 s along the time course of each session.^15^ Then, the functional connectivity within each sliding-time window was calculated between the seed channel and all other channels using the Pearson correlation method. For each stimulation session, functional connectivity variability (FCV) was calculated as the standard deviation of the correlation coefficient along time.^42^ For group analyses, the FCV of each correlation coefficient for each channel was averaged across subjects for each session. Paired t-tests were used to compare the FCV of each correlation coefficient between sessions, i.e., no tDCS versus low current tDCS, to determine whether the FCV between the seed channel and other channels in each stimulation session was significantly different (p<0.05). The channels with significant changes in FCV of the correlation coefficients with respect to the seed channels were plotted using the BrainNet Viewer software.^40^

#### 2.5.3 Graph theory analysis

Graph theory analysis was applied to investigate the changes in topographical patterns of functional networks across the entire cortical regions mapped by fNIRS. In our study, for each subject, we defined each fNIRS channel as a node, resulting in a total of 83 nodes. The edges $e_{ij}$ in the network were determined by setting a threshold, T, to the 83×83 Pearson’s correlation matrix values $r_{ij}$ by the following formula:^43^

$$e_{ij}=\{\begin{array}{cc} 1, & \text{if}\;|r_{ij}|>T\\ 0, & \text{otherwise} \end{array}.$$

A certain range of [$T_{\min}$ $T_{\max}$] for T was chosen so that $T_{\min}$ excluded weak and potentially nonsignificant connections while selecting the Pearson’s correlation coefficients that were significant and corresponded to p<0.05 in the channel-level correlation matrix.^44^ $T_{\max}$ was set based on the condition that the mean node degree $\overset{¯}{k}\geq 2\;\log$ (# of channels), which was equal to 8.8 for our setup.^44^^,^^45^ This value for $T_{\max}$ can be interpreted as the node being tested having connections with no less than 8.8 other nodes on average. Furthermore, this threshold value meant that the total number of edges in the network was no less than 365 (=83×8.8/2), equivalent to around 10% of the maximum number of edges possible (83×82/2=3403) in a network of 83 nodes.^46^^,^^47^ Once the statistically significant nodes and edges were identified, graph theory-based metrics were calculated by the Gretna software for each session.^43^

Clustering coefficient $C_{p}$ is the average of the clustering coefficients of all nodes. $C_{p}(i)$ of certain node i is defined as the following:^48^

$$C_{p}(i)=\frac{2N_{i}}{k_{\text{node}}(i)[k_{\text{node}}(i)-1]},$$

where $N_{i}$ denotes the number of existing connections among the neighbors of node i and $k_{\text{node}}$ represents the number of edges that are connected to node i.

Characteristic path length $L_{p}$ is defined as the average of the shortest path lengths between all pairs of nodes:^48^

$$L_{p}=\frac{1}{N(N-1)}\underset{i\neq j\in G}{\sum }d_{ij},$$

where N is the total node and $d_{ij}$ is the shortest path length between node i and node j.

The global efficiency $E_{\mathrm{glob}}$ and local efficiency $E_{\mathrm{loc}}$ are defined as the following:^49^

$$E_{\mathrm{glob}}=\frac{1}{N(N-1)}\underset{i\neq j\in G}{\sum }\frac{1}{d_{ij}},\;E_{\mathrm{loc}}=\frac{1}{N(N-1)}\underset{i\in G}{\sum }E(G_{i}),$$

where N is the total node, $d_{ij}$ is the shortest path length between node i and node j and $E(G_{i})$ is the global efficiency of the subgraph composed of the nearest neighbors of node i.

Small-world properties (clustering coefficient $C_{p}$ and characteristic path length $L_{p}$) and efficiency parameters (global efficiency $E_{\mathrm{glob}}$ and local efficiency $E_{\mathrm{loc}}$) were calculated by setting the threshold from $T_{\min}$ to $T_{\max}$ with a step size of 0.01.^44^^,^^45^^,^^50^ Then, the area under the curve (AUC) for each metric and stimulation session was computed. For group analyses, paired t-tests were used to compare the AUC for each metric and any significant changes (p<0.05) between stimulation sessions were identified.

## 3 Results

### 3.1 Functional Connectivity Analysis Using the Stimulated Cortical Region and Contralateral Cortical Region as the Seed

Paired t-tests for low current tDCS versus high current tDCS, and low current tDCS versus after high current tDCS were conducted when the seed was on channel 27 (Broca’s area) and channel 34 (Broca’s homologue area), and no significant changes in functional connectivity strength were found for either seed between stimulation conditions. These comparisons support our hypothesis that low current tDCS creates similar patterns to those of high current tDCS. In order to understand better how current intensity affected changes in connectivity patterns we focused all further analyses on comparisons with respect to the no tDCS condition, as described in the subsections here below.

#### 3.1.1 Using the stimulated left Broca’s area as the seed

**Fig. 4:**
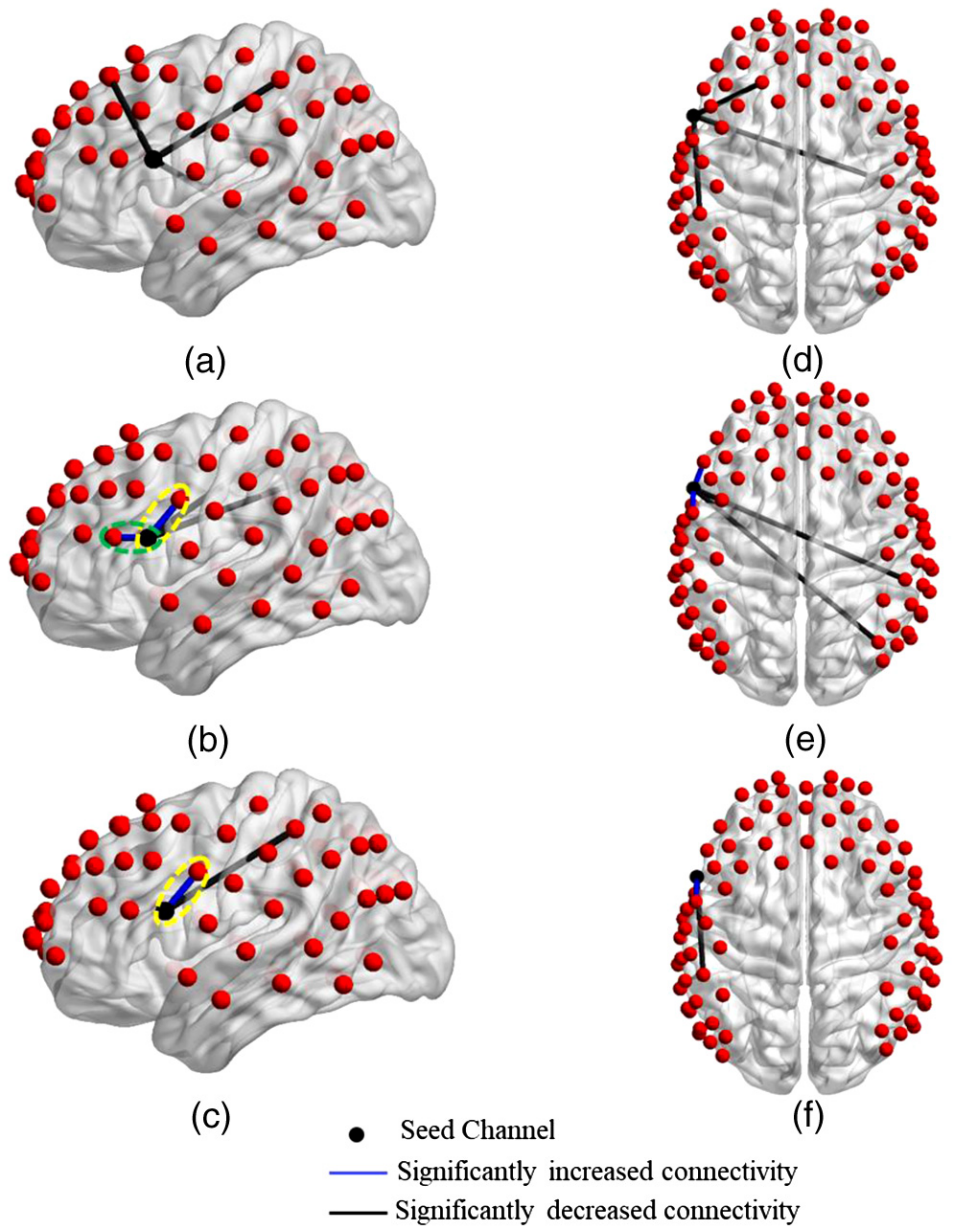
Changes in functional connectivity strength between the left Broca’s area (seed) and other cortical regions induced by different tDCS stimulation conditions. Group-level significant differences (p<0.05) in connectivity strength between pairs of detector locations for the no tDCS condition versus low current tDCS [(a) and (d)], versus high current tDCS [(b) and (e)], and versus after high current tDCS [(c) and (f)] stimulation conditions. Green ovals: detector pair locations showing significantly increased connectivity with the dorsolateral prefrontal area. Yellow ovals: detector pair locations showing significantly increased connectivity with the premotor and SMA areas.

Figure 4 shows the detector locations over cortical areas with significant changes in connectivity strength, as deduced from paired t-tests between the no tDCS condition versus each one of the other stimulation conditions (low current tDCS, high current tDCS, and after high current tDCS). Figures 4(a)–4(c) show lateral left views and Figs. 4(d)–4(f) show top views for each stimulation condition comparison. Figures 4(a) and 4(d) indicate that functional connectivity strength decreased significantly for longer distance connections with respect to the seed location at the left Broca’s area during the low current tDCS condition. Furthermore, Figs. 4(b) and 4(e) show that high current tDCS not only decreased significantly the connectivity strength with longer distance brain regions but also significantly increased the connectivity strength with nearby regions. This near-neighbor effect persisted during the after high current tDCS condition, as shown in Figs. 4(c) and 4(f), but the change in connectivity strength compared to the no tDCS condition was smaller compared to the high current tDCS condition. The increased connectivity strength between regions indicated by green and yellow ovals in Figs. 4(b) and 4(c) was most pronounced in the left dorsolateral prefrontal [green oval in Fig. 4(b), p=0.0010], premotor, and SMA areas [yellow oval in Figs. 4(b) and 4(c), p=0.0031 and p=0.0241], which regulate the planning, maintenance, and execution of speech.^51^^–^^54^

#### 3.1.2 Using the right Broca’s homologue area as the seed

**Fig. 5:**
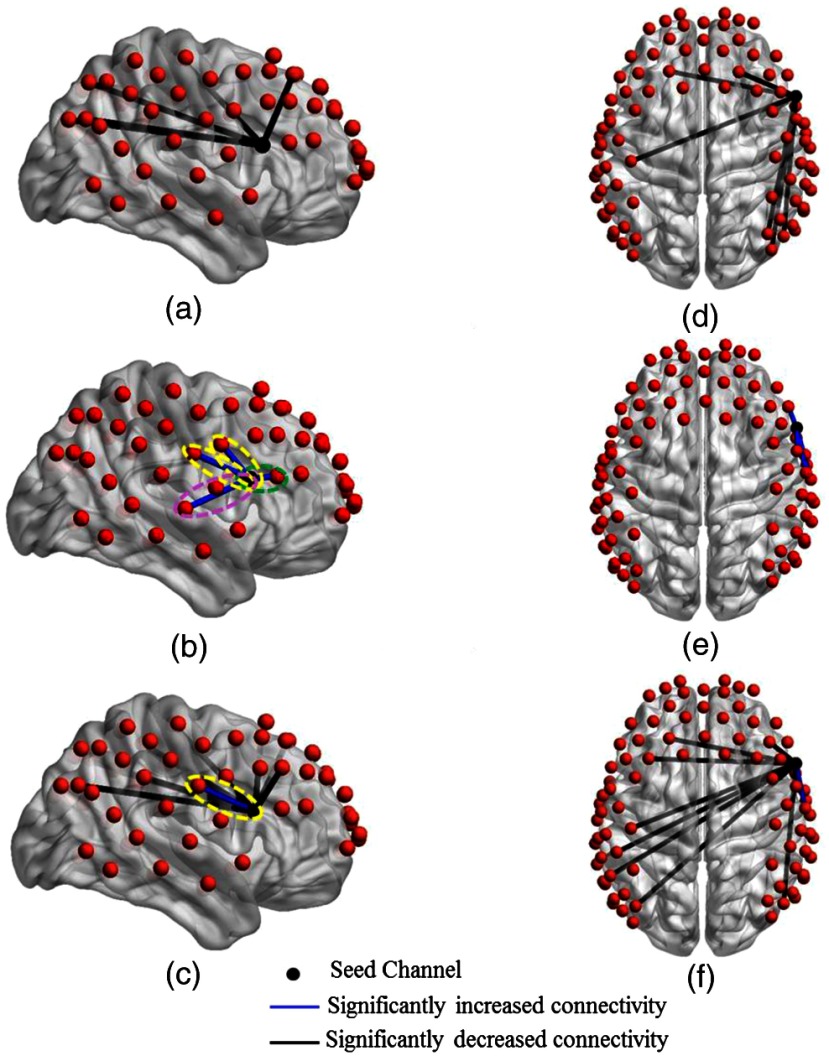
Changes in functional connectivity strength between the right Broca’s homologue (seed) and other cortical regions induced by different tDCS stimulation conditions. Group-level significant differences (p<0.05) in connectivity strength between pairs of detector locations for the no tDCS condition versus low current tDCS [(a) and (d)], versus high current tDCS [(b) and (e)], and versus after high current tDCS [(c) and (f)] stimulation conditions. Green ovals: detector pair locations showing significantly increased connectivity with the dorsolateral prefrontal area. Yellow ovals: detector pair locations showing significantly increased connectivity with premotor and SMA areas. Purple oval: detector pair locations showing significantly increased connectivity with the superior temporal gyrus.

Figure 5 shows detector locations over cortical areas with significant changes in connectivity strength using the right Broca’s homologue as the seed. These connectivity changes were deduced from paired t-tests between the no tDCS condition versus each one of the other stimulation conditions. Several qualitative similarities were observed in the connectivity strength changes when comparing to the left Broca’s area stimulation conditions: (1) low current tDCS induced significant decreases in connectivity strength for long-distance brain regions [Figs. 5(a) and 5(d)]; (2) high current tDCS brought in significant increases in connectivity strength for the nearby right dorsolateral prefrontal [green oval in Fig. 5(b), p=0.0045] and premotor and SMA areas [yellow ovals in Figs. 5(b), p=0.0329, and p=0.0241, respectively, and Fig. 5(c), p=0.0417]; (3) similar distance-dependent effects were seen for the after high current tDCS condition [Figs. 5(c) and 5(f)]. However, in contrast to the case of the left Broca’s area was the seed, high current tDCS also brought about a significant increase in connectivity strength with the right Broca’s homologue for detector location over another language-related cortical area, the superior temporal gyrus^55^ [purple oval in Fig. 5(b), p=0.0332]. This cortical region is part of Wernicke’s area and is related with prosody comprehension.^37^ In addition, we attribute the larger number of channels observed with significant connectivity changes compared to the case of stimulating left Broca’s area to the brain’s known asymmetry with more connections stemming from the right hemisphere of right-handed adults.^56^

In addition, Tables 1 and 2 list the Brodmann areas of channels with significantly decreased functional connectivity indicated in Figs. 4 and 5 for the three stimulation sessions compared to the no tDCS condition when the seed was on channels 27 and 34, respectively.

**Table 1:** Brodmann areas with significantly decreased steady-state functional connectivity when the seed was on channel 27 (left Broca’s area).

Seed: channel 27	Low current tDCS	High current tDCS	After high current tDCS
Ipsilateral hemisphere			
Brodmann area 5	FC decreased (p=0.0064)		FC decreased (p=0.0401)
Brodmann area 8	FC decreased (p=0.0416)		
Contralateral hemisphere			
Brodmann area 5		FC decreased (p=0.0420)	
Brodmann area 7		FC decreased (p=0.0187)	
Brodmann area 21	FC decreased (p=0.0311)		

**Table 2:** Brodmann areas with significantly decreased steady-state functional connectivity when the seed was on channel 34 (right Broca’s homologue).

Seed: channel 34	Low current tDCS	High current tDCS	After high current tDCS
Ipsilateral hemisphere			
Brodmann area 7	FC decreased (p=0.0250, 0.0201)		FC decreased (p=0.0442)
Brodmann area 8	FC decreased (p=0.0174)		FC decreased (p=0.0094)
Brodmann area 19	FC decreased (p=0.0068)		
Contralateral hemisphere			
Brodmann area 3	FC decreased (p=0.0194)		FC decreased (p=0.0277)
Brodmann area 5			FC decreased (p=0.0496, 0.0241)
Brodmann area 7			FC decreased (p=0.0331)
Brodmann area 8	FC decreased (p=0.0479)		FC decreased (p=0.0094, 0.0087)

### 3.2 Time-Variant Functional Connectivity using the Stimulated Left Broca’s Area as Seed

**Fig. 6:**
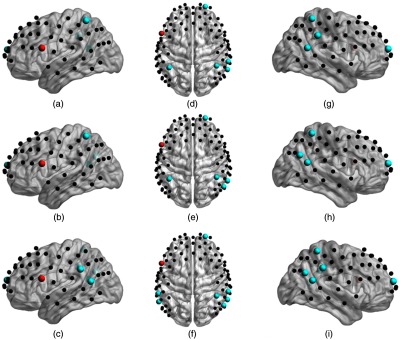
Brain cortical regions of significant increased FCV (blue dots, FCV, p<0.05) with stimulated cortical region (red dots) induced by different tDCS stimulation conditions: (a), (d), (g) no tDCS versus low current tDCS; (b), (e), (h) no tDCS versus high current tDCS; and (c), (f), (i) no tDCS versus after high current tDCS.

Figures 6(a)–6(c) illustrate the detector locations over cortical regions (blue dots) with significantly increased FCV of the time-variant functional connectivity when the left Broca’s area was the stimulated cortical region (red dots). Each row in that figure corresponds to a distinct stimulation condition, namely low current tDCS, high current tDCS, and after high current tDCS. The cortical regions with increased FCV were similar for all three tDCS conditions: left Wernicke’s area, right Wernicke’s area, and right frontopolar area. Similar results were also found when using the right Broca’s homologue as the seed. The left and right Wernicke’s areas and the left frontopolar area also showed significantly increased FCV for all three tDCS sessions. Due to their similarity to the findings for the seed over the left Broca’s area, we do not show these results for brevity. These cortical regions belong to higher order brain regions (including language processing areas and frontopolar prefrontal cortex)^57^^,^^58^ and are remote to the seed regions. These FCV findings are in contrast to the above steady-state connectivity strength findings that mostly highlighted local connectivity strength increases.

Tables 3 and 4 list Brodmann areas of channels with significantly increased FCV for the three stimulation sessions compared to the no tDCS condition when the seed was on channels 27 and 34, respectively.

**Table 3:** Brodmann areas with significantly increased FCV when the seed was on channel 27 (left Broca’s area).

Seed: channel 27	Low current tDCS	High current tDCS	After high current tDCS
Ipsilateral hemisphere			
Brodmann area 22			FCV increased (p=0.0189)
Brodmann area 40	FCV increased (p=0.0468)	FCV increased (p=0.0358)	FCV increased (p=0.0421)
Contralateral hemisphere			
Brodmann area 10	FCV increased (p=0.0002)	FCV increased (p=0.0085)	FCV increased (p=0.0001)
Brodmann area 22	FCV increased (p=0.0321)	FCV increased (p=0.0303)	FCV increased (p=0.0232)
Brodmann area 39		FCV increased (p=0.0383)	FCV increased (p=0.0033)
Brodmann area 40	FCV increased (p=0.0356, 0.0044)	FCV increased (p=0.0021)	FCV increased (p=0.0335, 0.0111)

**Table 4:** Brodmann areas with significantly increased FCV when the seed was on channel 34 (right Broca’s homologue).

Seed: channel 34	Low current tDCS	High current tDCS	After high current tDCS
Ipsilateral hemisphere			
Brodmann area 39	FCV increased (p=0.0422)	FCV increased (p=0.0420)	FCV increased (p=0.0013)
Brodmann area 40			FCV increased (p=0.0278)
Contralateral hemisphere			
Brodmann area 10	FCV increased (p=0.0405)	FCV increased (p=0.0151)	FCV increased (p=0.0073)
Brodmann area 39	FCV increased (p=0.0230)	FCV increased (p=0.0195)	
Brodmann area 40			FCV increased (p=0.0426)

In addition, paired t-tests were conducted for low current tDCS versus high current tDCS, and low current tDCS versus after high current tDCS. There were no significant changes on FCV when the seed was on channel 27 or channel 34. These results are consistent and our findings from comparisons with the no tDCS condition that low current tDCS creates similar FCV patterns to those of high current tDCS.

### 3.3 Graph Theory Analysis

As described in Sec. 2.5.3, a certain range of functional connectivity thresholds [0.3, 0.59] with the step of 0.01 was chosen to perform graph theory analysis.

**Fig. 7:**
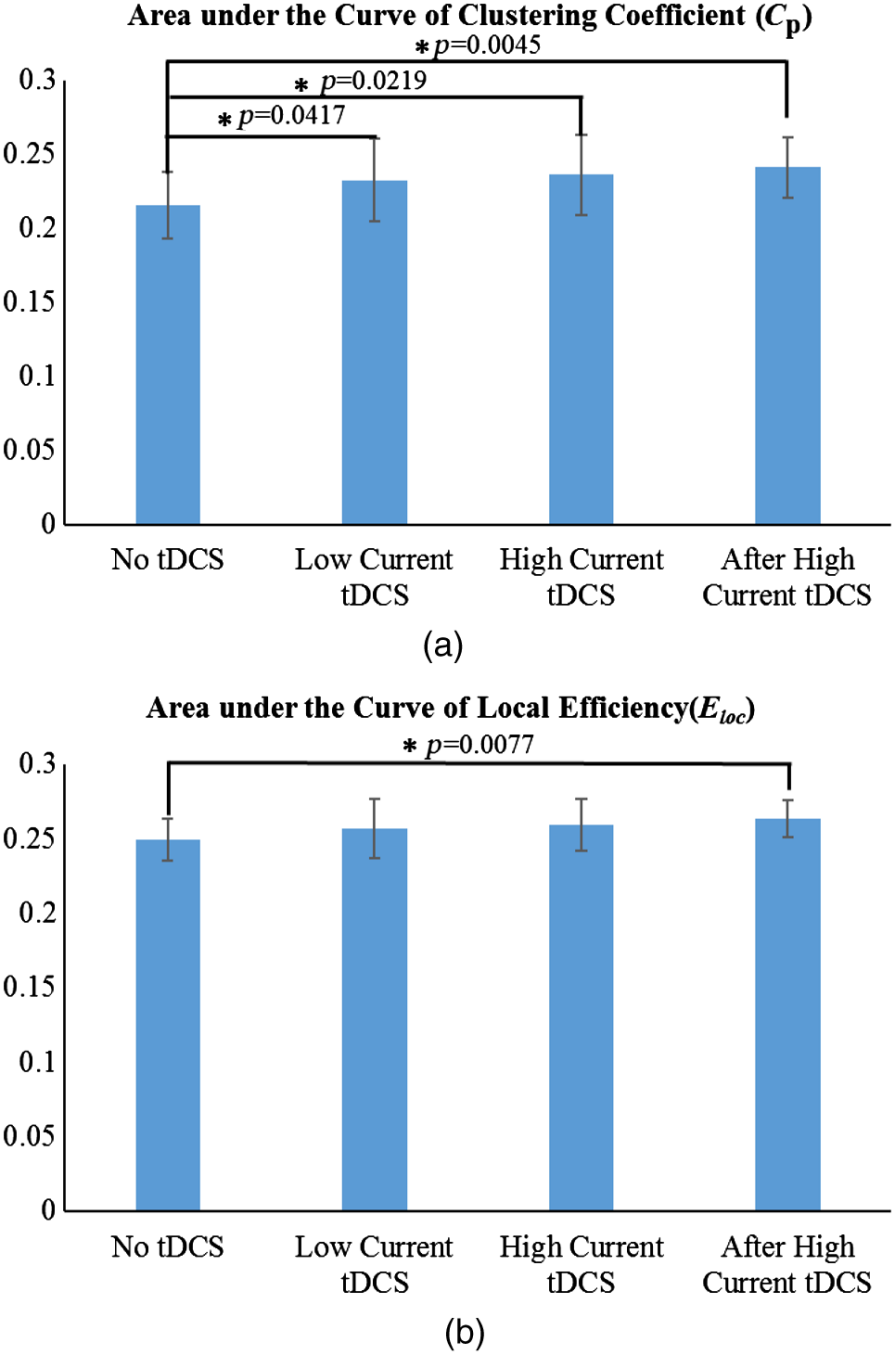
The differences of mean areas under the curve (AUC) for (a) clustering coefficient ($C_{p}$) and (b) local efficiency ($E_{\mathrm{loc}}$) computed for the no tDCS, low current tDCS, high current tDCS, and after high current tDCS conditions. The asterisk indicates significant differences (p<0.05). Error bars correspond to one standard deviation to the mean across subjects.

Figures 7(a) and 7(b) illustrate the AUC for the cortical network clustering coefficient ($C_{p}$) and local efficiency ($E_{\mathrm{loc}}$), respectively, with the AUC being integrated as a function of threshold T^43^ for the different stimulation conditions. It was found that the AUC for both $C_{p}$ and $E_{\mathrm{loc}}$ had similar trends, showing an increase in going from the no tDCS to the after high current tDCS condition. However, significant changes (p<0.05) were found between the no tDCS condition and all other stimulation conditions for $C_{p}$, whereas for $E_{\mathrm{loc}}$, the only significant change was between the no tDCS and the after high current tDCS conditions. The significant changes in the AUC of $C_{p}$ for all stimulation conditions relative to baseline reflect an increase in the number of neighboring channel connections, even for low stimulation currents and after the high current tDCS was turned off. In addition, the significant change in the AUC for $E_{\mathrm{loc}}$ suggests that the after high current tDCS condition enhanced the formation of network clusters, which is consistent with more efficient communication between nodes within the same immediate neighborhood.^59^^,^^60^

Furthermore, no significant differences were found in the AUC of $C_{p}$ for low current tDCS versus high current tDCS (p=0.604), and low current tDCS versus after high current tDCS (p=0.177). Also, no significant differences were found in the AUC of $E_{\mathrm{loc}}$ for low current tDCS versus high current tDCS (p=0.601), and low current tDCS versus after high current tDCS (p=0.120). These results are consistent and our findings from comparisons with the no tDCS condition that low current tDCS creates similar graph theory derived metrics to those of high current tDCS.

## 4 Discussion

This study systematically explored by use of resting state fNIRS the impact of two different anodal tDCS exposures applied over the left Broca’s area, on functional connectivity reorganization. A lower tDCS current (low current tDCS) was applied to create transient hemodynamic changes, whereas a higher current (high current tDCS) mimicked a current dose that is typical of tDCS interventions reported in the literatures.^61^^,^^62^ The purpose of this work was to test how tDCS modulated connectivity in the immediate neighborhood of the anodal stimulation location over left Broca’s area versus the connectivity to distant cortical regions involved in language processing. A second goal was to explore whether lower currents could reproduce qualitatively the connectivity patterns seen for typical intervention currents, which would enable the lower currents to serve as predictors of connectivity responses to the higher currents.

### 4.1 tDCS Increased Steady-State Functional Connectivity in the Immediate Neighborhood of the Stimulation Location

Seed-based analysis revealed distinct differences in functional connectivity patterns between low current tDCS, high current tDCS, and within 10 min after high current tDCS. Specifically, low current tDCS was more likely to suppress the connections with long distance brain regions, while high current tDCS encouraged increased local connectivity. Interestingly, during the after high current tDCS session, a return to the suppression of long-distance connections was seen, combined with the short-distance increased connectivity seen during the immediately preceding high current tDCS session. It is hypothesized that anodal tDCS-induced subthreshold neuronal depolarization, which reduced the amount of excitatory input required, resulting in the excitation of the stimulated cortical areas near the anodal electrode.^6^^,^^7^ The local increase of spontaneous activity might have decreased the neuronal signal to noise ratio, and consequently decreased the synchronization with other remote brain regions,^39^^,^^63^ which could help explain the observed reduction in connectivity with those regions. Similar results with respect to changes in local versus long-distance connectivity were reported in prior work^63^ for anodal tDCS, corresponding to high current tDCS in this work, for stimulation over the left primary motor area. In this work, the brain regions with the most pronounced increases in functional connectivity strength were the dorsolateral prefrontal, premotor, and SMA areas, known to be a part of the language processing and production network.^51^^–^^54^ These results were consistent with prior studies reporting on dual tDCS over the Broca’s areas,^13^^,^^14^ where it was illustrated that tDCS resulted in significantly increased functional connectivity with the premotor and SMA regions. High current tDCS also brought about a significantly increased functional connectivity between the right Broca’s homologue and the right superior temporal gyrus, which are known to be responsible for prosody comprehension function.^37^ For the two channels with persistently increased connectivity strength during the after high current tDCS sessions [Figs. 4(c) and 5(c)], the increase in connectivity strength during the high current tDCS session was larger than other increased connections [Figs. 4(b) and 5(b)], with the latter going back to prestimulation levels during the after high current tDCS session [Figs. 4(c) and 5(c)]. Our results suggest that anodal stimulation over the left Broca’s area prepares the task-related language areas by enhancing functional connectivity with these cortical regions. We hypothesize that enhanced baseline connectivity strength between language areas contributed to the beneficial effects of anodal tDCS stimulation previously seen for picture naming performance^12^ and verbal fluency^64^ in healthy subjects. Similarly, these findings might be helpful to explain the beneficial effects of anodal stimulation over the affected hemisphere in stroke aphasia patients.^8^^,^^65^^,^^66^ In addition, low current tDCS could not predict the increased functional connectivity in the immediate neighborhood of the stimulation location induced by high current tDCS. However, for the decreased functional connectivity in the remote regions of the stimulation location, seen in Tables 1 and 2 as a whole, low current tDCS did not produce significant changes for the same Brodmann areas as high current tDCS, though it showed changes for the same 5/7 Brodmann areas as the after high current tDCS condition. These results, taken together with Figs. 4 and 5, indicate that low current tDCS is at best a weak qualitative predictor of the connectivity change patterns seen after high tDCS and no further statistical comparisons were performed to quantify its predictive ability.

### 4.2 tDCS Increased Functional Connectivity Variability with High-Order Cognitive Cortical Regions

In contrast to steady-state functional connectivity that focuses on connectivity strength, FCV focuses on the variation of the connectivity strength of transient states. It was observed (Fig. 6) that all of low current tDCS, high current tDCS, and after high current tDCS sessions induced a significant increase in FCV between Wernicke’s area and the right frontopolar areas after anodal stimulation of the left Broca’s area. It is known that the left Wernicke’s area is involved in comprehension or understanding of written and spoken language, while its homologous area has a role in the processing and resolution of subordinate meanings of ambiguous words.^67^ Also, the right frontopolar area is associated with memory function^68^ and both Wernicke’s and frontopolar areas belong to high-order language and memory processing networks.^57^^,^^58^ Previous studies reported that higher-order brain networks show large variability in connection strength over time.^16^^,^^69^ Our results suggest that tDCS resulted in larger FCV in these regions, which we interpret as them having a higher degree of flexibility for performing higher-order language processing tasks after stimulation.^16^^,^^70^ The more variable functional connectivity in these connections potentially reflects tDCS facilitating the emergence of a large-scale network with flexible capability in functional coordination between language and memory systems. Besides, as shown in Tables 3 and 4, low current tDCS showed FCV increases in the same 7/8 Brodmann areas during high current tDCS and the same 6/10 areas during the after high current tDCS session. The majority of Brodmann areas missed (mainly Brodmann area 40 when the seed on channel 34) in the after high current tDCS session were neighboring to the areas that existed during both low current tDCS and high current tDCS (Brodmann area 39 when the seed was on channel 34). Therefore, low current tDCS presented similar results with increased FCV in high-order cognitive cortical regions, thus offering the possibility to serve as a qualitative predictor of patterns in FCV changes induced during the high current tDCS and after high current tDCS conditions.

### 4.3 tDCS Enhanced the Small-World Features of the Cortical Network Globally

In contrast with seed-based functional connectivity, graph theory analysis quantified network connectivity changes induced by tDCS across the entire cortical area mapped by fNIRS. tDCS induced an increase in the density of local connections between detector channels across the entire cortical network that was quantified by the network’s small-world features. These network features were the characteristic path length ($L_{p}$), the clustering coefficient ($C_{p}$), global efficiency ($E_{\mathrm{gb}}$), and the local efficiency ($E_{\mathrm{loc}}$).^49^^,^^71^^,^^72^ Two of these small world properties were enhanced by tDCS: $C_{p}$ became significantly higher for all stimulation sessions, whereas $E_{\mathrm{loc}}$ became significantly higher only in the after high current tDCS session. The enhancement of small-world features after tDCS is interpreted as the cortical networks becoming more segregated^71^ and clustered with more locally efficient communication and higher fault tolerance.^59^ The graph theory analysis findings were consistent with our seed-based functional connectivity results, where the seed areas were tightly connected with local cortical regions and desynchronized with more distant regions. In addition, changes in graph theory metrics induced by low current tDCS were qualitatively similar to the ones induced by high current tDCS, which indicates that low current tDCS could be used as a tool for studying qualitatively the patterns brought about high current tDCS.

### 4.4 Limitations of the Study and Future Work

A few potential limitations should be noted for this study. First, the gender distribution was not even (2 females versus 11 males). We conducted the same analyses when excluding the two female subjects and the vast majority of results did not change, with three exceptions. First, when comparing the no tDCS versus low current tDCS conditions, there was one more channel located in the contralateral BA21 and one channel located in the ipsilateral BA8 with significantly decreased functional connectivity with the seed on left Broca’s area and right Broca’s homologue, respectively (p=0.0334 and p=0.0197, respectively). Second, the AUC of $E_{\mathrm{loc}}$ had significant changes when comparing the no tDCS versus low current tDCS (p=0.0462) and the no tDCS versus high current tDCS (p=0.0268) conditions for the male population only. Third, when comparing the low current tDCS versus high current tDCS conditions, there was one channel located in the ipsilateral dorsolateral prefrontal BA9 with significantly increased functional connectivity when the seed was on the left Broca’s area (p=0.0378). However, all these changes were marginal and cannot be attributed conclusively to gender effects since we only had two female subjects. Previous MRI studies^73^^,^^74^ reveal significant gender differences within small regions of interest in language networks during semantic language processing, of which the middle temporal gyrus and pars opercularis are at accessible depths for fNIRS too. To reach more certain assertions about the effect of gender on the response to tDCS, it would be important to include more female subjects and larger numbers of subjects overall in a future study. An additional limitation was that even though low current tDCS could qualitatively predict some aspects of changes in functional connectivity patterns induced by high current tDCS, those changes were not linked to meaningful behavioral changes in this study. Therefore, in a future study, the performance of language tasks, such as picture naming accuracy^8^^,^^19^ and response time,^9^^,^^12^^,^^19^ should be included when low current tDCS and high current tDCS are applied. Finally, the lack of resemblance between connectivity changes seen during low current tDCS and high current tDCS indicates that it is not feasible to use lower currents as a rapid qualitative predictor of patterns seen at higher currents, in the resting state at least. This finding is not surprising since the effect of local connectivity increase is known to only occur at higher currents that can create depolarization of the resting membrane potential and increasing neuronal firing rates.^5^ Nevertheless, the qualitative resemblance of connectivity change patterns seen for low current tDCS versus after high current tDCS, both for steady-state and time-dependent connectivity metrics, shows some promise for the hypothesis that lower currents could be used to predict qualitatively the connectivity change patterns seen after higher current, clinically relevant, stimulation intensities. Nevertheless, further work is needed to verify the validity of this hypothesis.

## 5 Conclusion

Our work demonstrates the feasibility of using resting-state fNIRS to study cortical network reorganization induced by tDCS in the language processing cortical networks of healthy subjects. Seed-based functional connectivity, graph theory analysis, and time-variant functional connectivity were used to track changes in cortical functional connectivity for these networks. Seed-based connectivity and graph theory analyses revealed increased local and decreased remote functional connectivity induced by tDCS. At the same time, time-variant functional connectivity changes suggested that tDCS increased FCV of remote connections. In addition, low current tDCS produced qualitatively similar patterns of increases in clustering coefficient and FCV, but did not produce patterns of increased local connectivity observed during and immediately after high current tDCS. The qualitative similarity of connectivity patterns seen between the low current tDCS and after high current tDCS conditions hints at the possibility of using multiple electrode placements with low stimulation currents in a single protocol to gain insight into what these changes could be at higher currents for each subject. Nevertheless, the validity of this approach needs to be tested in further work involving stimulation during performance of a task so that connectivity changes could be associated with observable task performance changes.
